# Real-time fluorescence and colorimetric identification of bulbus fritillariae using recombinase assisted loop-mediated isothermal DNA amplification (RALA)

**DOI:** 10.3389/fpls.2022.948879

**Published:** 2022-07-28

**Authors:** Yinghua Wei, Sheng Ding, Gangyi Chen, Juan Dong, Feng Du, Xin Huang, Xin Cui, Rong Chen, Zhuo Tang

**Affiliations:** ^1^Natural Products Research Center, Chengdu Institute of Biology, Chinese Academy of Sciences, Chengdu, Beijing, China; ^2^School of Ethnic Medicine, Chengdu University of Traditional Chinese Medicine, Chengdu, China; ^3^State Key Laboratory of Southwestern Chinese Medicine Resources, The Ministry of Education Key Laboratory of Standardization of Chinese Herbal Medicine, Chengdu University of Traditional Chinese Medicine, Chengdu, China

**Keywords:** bulbus fritillariae, isothermal amplification, molecular identification, colorimetric detection, herbal medicine, recombinase assisted loop-mediated isothermal DNA amplification

## Abstract

Bulbus Fritillariae (BF) is a kind of herbal medicine widely used in many countries including China, Japan, Korea, and so on. Among the known BF medicinal materials, Bulbus Fritillariae cirrhosae (BF cirrhosae) was reported to have the best curative effect. Due to the limited resources of BF cirrhosae, a lot of adulterants have emerged in the market, impairing the market order, resource development, and above all, clinical efficacy. Here, a novel nucleic acid amplification technique, Recombinase Assisted Loop-mediated isothermal DNA Amplification (RALA), was used to establish a real-time fluorescence isothermal molecular authentication method for five commonly used BF drugs. Moreover, this RALA-based assay can also be developed as a colorimetric detection method for on-site detection. Both real-time fluorescence and visual methods could detect as low as 0.1% genuine targets in the mixed samples. In summary, we report an isothermal detection system for five kinds of BF circulating in the market, providing a new choice for the molecular identification of BF drugs and showing promise in the laboratory testing as well as field identification of other herbal medicines.

## Graphical Abstract

Workflow of RALA-based bulbus fritillariae identification. 
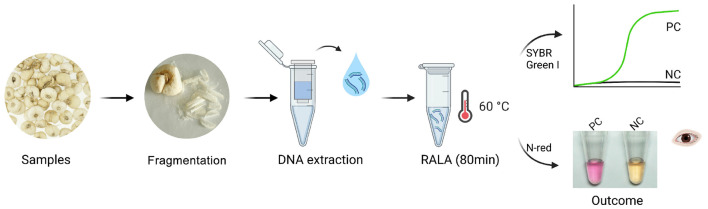

## Introduction

As a famous herbal medicine, Bulbus Fritillariae (BF) derives from the dried bulbs of various species of the genus *Fritillaria*. BF was reported to have the function of clearing away heat and moistening lungs, eliminating phlegm, and relieving cough (Xu et al., 2016; Editorial Committee of Pharmacopoeia of the People’s Republic of China, 2020). However, the usage and dosage of different BF drugs should be administrated dissimilarly as they differ from each other in chemical characteristics and pharmacological effects (Li et al., 2006), although sharing similar morphology traits and smell (Chen et al., 2018). Among all these kinds of BF drugs, BF cirrhosae has the most extraordinary curative effect and highest medicinal value, which, together with the scarcity of wild resources and long growth cycle, contribute to its high price (Cunningham et al., 2018). Under such circumstances, many BF cirrhosae sold in the market are adulterated by other kinds of BF, severely disturbing the market order, and affecting the clinical efficacy. Therefore, an accurate identification method for BF drugs is urgently needed for ensuring authenticity, clinical medication safety, and traceability.

Traditional identification methods for BF drugs include morphologic detection, microscopical identification (Wang et al., 2006), physical and chemical tests (Xu et al., 2019), chromatographic and spectral analysis (Cui et al., 2018; Luo et al., 2018). Due to its high accuracy and specificity, DNA-based molecular biology techniques have been becoming increasingly popular in the identification of herbal medicines as well as processed herbal products (Chen et al., 2010, 2012, 2017). Among the various DNA identification methods, polymerase chain reaction (PCR) is the first and remains the most popular DNA amplification technology for distinguishing various kinds of herbal medicines (Han et al., 2018; Chen et al., 2019), while isothermal DNA amplification methods are getting more popular because of their potential use in field-testing (Ma et al., 2018; Lan et al., 2019; Lee and Hxiao, 2019). Loop-mediated isothermal amplification (LAMP) is one of the most commonly used isothermal DNA amplification methods, which requires four to six primers, theoretically increasing the difficulty of primer design as well as the probability of non-specific amplification caused by primer-dimer (Rolando et al., 2020). Apart from conventional reporting systems that require uncapping operation, visualized reporting has been attracting considerable attention in on-site detection as it could efficiently decrease the risk of carry-over contamination. One-pot colorimetric assays always take the advantage of some amplification characteristics of isothermal amplification methods, such as the consumption of Magnesium ion and the pH value drop (Tomita et al., 2008; Goto et al., 2009; Tanner et al., 2015). So far, many indicators including metal ion indicators and pH-sensitive indicators have been successfully applied to isothermal detection (He et al., 2018; Ding et al., 2019).

In an effort to overcome the shortcomings of LAMP, an isothermal amplification method called RALA (Recombinase Assisted Loop-mediated isothermal DNA Amplification) was developed by our group (Chen G. Y. et al., 2020). The primer design of RALA requires only two to four primers, efficiently reducing the chance of non-specific amplification caused by primer dimers compared to LAMP. Additionally, the introduction of recombinase into the reaction may further improve the specificity of RALA (Shigemori et al., 2005). Therefore, we developed a specific DNA identification system using RALA to distinguish various BF drugs. In addition to real-time testing, a one-pot colorimetric reporting system was also established in this study.

## Materials and methods

### Preparation of the deoxyribonucleic acid sample

BF cirrhosae (bulbus of *F. cirrhosa* D. Don, *F. unibracteata* Hsiao et K. C. Hsia, *F. delavayi* Franch., *F. taipaiensis* P. Y. Li, *F. przewalskii* Maxim. and *F. unibracteata* Hsiao et K. C. Hsia var. *wabuensis* (S. Y. Tanget S. C. Yue) Z. D. Liu, S. Wang et S. C. Chen), BF ussuriensis (bulbus of *F. ussuriensis* Maxim.), BF pallidiflora (bulbus of *F. pallidiflora* Schrenk and *F. walujewii* Regel), BF thunbergii (bulbus of *F. thunbergii* Miq.) and BF hupehensis (bulbus of *F. hupehensis* Hsiao et K. C. Hsia) were gifted by Chengdu Institute Food and Drug Control and authenticated by DNA barcoding using ITS as a marker gene. The DNA of all BF samples was extracted using the plant DNA isolation Kit (Foregene Co., Ltd., Chengdu, China) according to the manufacturer’s instructions. The concentration of DNA was determined by the ultra-micro ultraviolet spectrophotometer ND5000 (BioTeke Co., Ltd., Beijing, China) and stored at 4°C. Finally, 1 μL DNA sample was directly added to the RALA system.

### Polymerase chain reaction

The total volume of the PCR reaction mixture was 20 μL, containing 1 × EasyTaq buffer (20 mM Tris–HCl (pH 8.4), 20 mM KCl, 2 mM MgSO_4_, and 10 mM (NH_4_)_2_SO_4_, 0.2 mM dNTPs, 3 U of EasyTaq polymerase, 0.2 μM each primer (Supplementary Table 1), and 1 μL DNA template. The PCR reaction mixture was placed in a C1000TM Thermal Cycler PCR (Bio-Rad Laboratories, United States) and denatured at 94°C for 2 min, followed by 35 thermal cycles (denaturation at 94°C for 5 s, renaturation at 56–60°C for 5 s, extension at 72°C for 20 s), and extension at 72°C for 2 min. The results were analyzed through 2% agarose gel and sequenced by BBI [Sangon Biotech (Shanghai) Co., Ltd., Shanghai, China].

### Recombinase assisted loop-mediated isothermal DNA amplification primer design

The RALA primers included a pair of inner primers and one (or two) loop primers were designed according to the specific site of the ITS sequence or the P16 gene sequence. All oligonucleotides were synthesized and purified by BBI, and the purification level was PAGE. The RALA primer sequences of 5 kinds BF were shown in Supplementary Table 2.

### Real-time recombinase assisted loop-mediated isothermal DNA amplification reaction

The total volume of the RALA reaction mixture was 25 μL, including 20 mM Tris–HCl (pH 8.8), 60 mM KCl, 4 mM Mg SO_4_, 10 mM (NH_4_)_2_SO_4_, 0.1% Triton X-100, 0.8 M betaine, 0.8 mM dNTPs, 1.6 μM inner primers (FIP and BIP), 0.8 μM loop primers (LF and LB), 0.24 U/μL *Bsm* polymerase, 0.6 mM ATP, 6 ng/μL *Tth*RecA, 0.2 × SYBR Green I, and 1 μL DNA sample. Each group of primers amplified five BF at the same time. The reaction mixture was placed in the PikoReal Real-Time PCR System (Thermo Fisher Scientific, Waltham, MA, United States) at 60°C for 80 min. The results could be analyzed through the real-time collected fluorescence signals and 2% agarose gel.

### Sensitivity of the real-time recombinase assisted loop-mediated isothermal DNA amplification

Taking BF cirrhosae as the target, the analytical sensitivity of RALA was investigated by using a 10-fold serial dilution of pure genomic DNA (25 ng/μL –2.5 × 10^–5^ ng/μL) and the mixed templates which contained different percentages of BF cirrhosae in the mixed genome with maize (100, 10, 1, 0.1, 0.01, and 0%, respectively, regards to total 25 ng/μL of genomic DNA).

### Visualization of recombinase assisted loop-mediated isothermal DNA amplification detection

N-red was used as the chromogenic reagent of RALA detection in the establishment of the colorimetric analysis platform for five BF. The colorimetric system is based on real-time RALA system, in which SGI is replaced by 200 μM N-red, and the reaction was incubated at 60°C for 80 min. The results of visual color reaction were captured with Canon 600D camera (Canon, Japan) under the background of white paper.

## Results

### The mechanism of bulbus fritillariae identification using recombinase assisted loop-mediated isothermal DNA amplification

**FIGURE 1:**
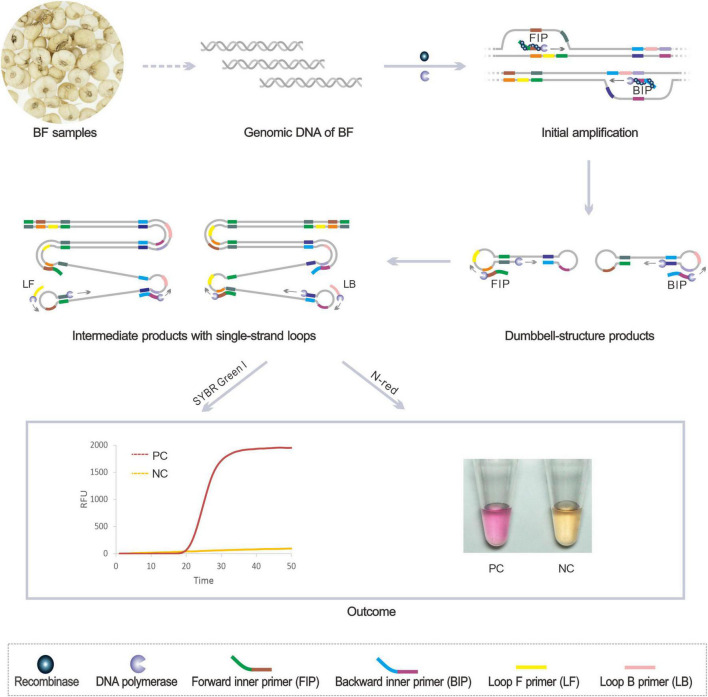
Schematic diagram of the Recombinase Assisted Loop-mediated isothermal DNA Amplification detection method of BF samples. PC, positive control with DNA target; NC, negative control without the target.

The genome sequence-based molecular detection leverages the specific gene fragment as the amplification target to accurately identify the medicinal plants of related species. After decades of practice, ITS sequence (China Plant BOL Group et al., 2011; Shi et al., 2020), rbcL sequence (Chase et al., 2005), and matK sequence (Hollingsworth et al., 2011) have been proved to be useful DNA barcodes for molecular identification of medicinal plants. Figure 1 illustrates the principle of the BF identification using our newly invented RALA. Firstly, the recombinase *Tth RecA* binds with the inner primers FIP and BIP to form the nucleoprotein complex and open the target double-strand DNA (dsDNA) at the consensus region. Next, the primers anneal to the complementary strand and are extended by the DNA polymerase with strand displacement ability. After the initial extension and strand displacement of the inner primer, the dumbbell-like amplicon is generated and self-primed for further extension. Moreover, the new inner primers can anneal to the dumbbell-like amplicon to induce the next round of extension. Besides, the loop primers LF and LB were designed to anneal to the single-strand loop domain of the intermediated products, which can further improve the amplification rate of RALA. The whole amplification process can be completed at a constant temperature of 60°C and the products of RALA amplification were considerable stem-loop DNAs with inverted target repeats. For different analysis needs, both the real-time fluorescence and colorimetric reporting system were established to present the results (which will be introduced in detail in the two chapters of *Real-time RALA detection of BF cirrhosae* and *Colorimetric detection of BF cirrhosae*).

### Real-time recombinase assisted loop-mediated isothermal DNA amplification detection of bulbus fritillariae cirrhosae

**FIGURE 2:**
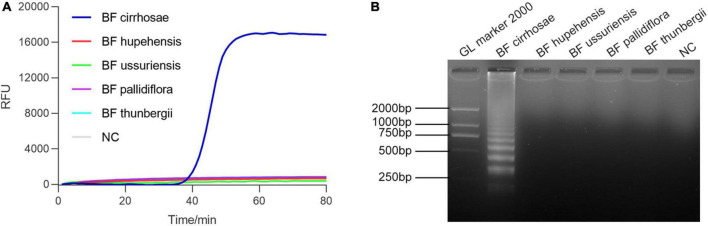
The specificity of the designed primers (BF cirrhosae-6). **(A)** Discrimination plot of real-time Recombinase Assisted Loop-mediated isothermal DNA Amplification (RALA) for BF cirrhosae using SYBR Green I. **(B)** Validation of real-time RALA amplification by agarose gel electrophoresis.

Bulbus Fritillariae cirrhosae is the bulbus of six species of Fritillariae: (1) *F. cirrhosa* D. Don; (2) *F. unibracteata* Hsiao et K. C. Hsia; (3) *F. przewalskii* Maxim.; (4) *F. delavayi* Franch.; (5) *F. taipaiensis* P. Y. Li; (6) *F. unibracteata* Hsiao et K. C. Hsia var. *wabuensis* (S. Y. Tang et S. C. Yue) Z. D. Liu, S. Wang et S. C. Chen). Fortunately, the ITS sequences of the six source plants of BF cirrhosae are identical in large part, and there are multiple base sites different from the other BF (Supplementary Figure 1B). To develop the RALA-based detection method for BF cirrhosae, the ITS sequence region of the six kinds of BF cirrhosae was selected for primer design. Several sets of specific primers of BF cirrhosae were designed according to the alignment result of ITS sequences (Supplementary Table 3) and evaluated by amplifying the genomic DNA of various BF drugs (Supplementary Figure 2). As shown in Figure 2A, primer set BF cirrhosae-6 shows the highest specificity as the rising fluorescence curve only occurred when amplifying the genomic DNA of BF cirrhosae while the fluorescence curves remained flat when amplifying other BF genomic DNA, indicating no cross-reaction when amplifying non-target samples. The amplification products of Figure 2A were further verified by agarose gel electrophoresis. As depicted in Figure 2B, the typical band only appeared in amplification products of BF cirrhosae. These results demonstrated that the RALA-based assay could efficiently distinguish BF cirrhosae from their related kinds. It is worth mentioning that equal proportion mixed samples of six BF cirrhosae was used as BF cirrhosae samples for the described specificity detection as the sequence of the target fragment is almost identical among these six kinds of BF cirrhosae (Supplementary Figure 1). In order to verify it, the optimal primer set was used to detect all these six BF cirrhosae and the mixed samples, respectively. The rising fluorescence curves in Supplementary Figure 3 show that this primer set is able to detect all six kinds of BF cirrhosae and the mixed samples with a similar efficiency.

### Sensitivity of the real-time recombinase assisted loop-mediated isothermal DNA amplification

**FIGURE 3:**
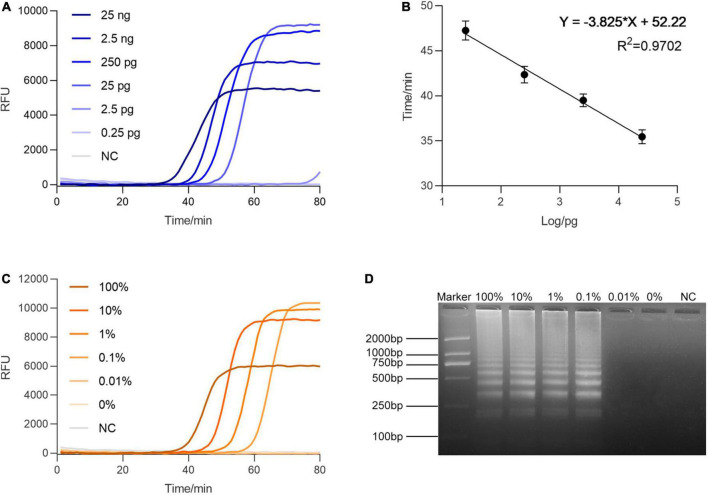
Sensitivity analysis of real-time Recombinase Assisted Loop-mediated isothermal DNA Amplification (RALA). **(A)** Sensitivity of the method in the range of 25 ng–2.5 pg of BF cirrhosae genome. **(B)** The standard curve of the linear relation between the Tq value and the logarithm of the DNA quality in 25 ng–25 pg. The error bars are the standard deviation of two repetitive measurements. **(C)** Sensitivity analysis of real-time RALA with different percentages (100, 10, 1, 0.1, 0.01, 0%) of BF cirrhosae templates. **(D)** Gel electrophoresis analysis of the different percentages (100, 10, 1, 0.1, 0.01, 0%) of BF cirrhosae amplification results.

After the specificity examination, we sought to evaluate the sensitivity of this method. The serially diluted genomic templates of BF cirrhosae were used to determine the minimum detectable quantity of BF by RALA. As presented in Figure 3A, the reactions with template concentrations ranging from 25 ng to 2.5 pg manifested the rising fluorescence curves within 80 min, suggesting RALA could efficiently detect down to about 25 pg of the target template. To explore the quantitative ability of the real-time RALA assay, the Tq value defined as the reaction time when the fluorescence intensity exceeds the arbitrary threshold was introduced to the assay. As shown in Figure 3B, the Tq value linearly correlates with the target concentration (*R*^2^ = 0.9702), indicating that this method has the potential to realize quantitative detection. To evaluate the sensitivity of the method in mixed samples, 25 ng of the mixed genome containing different ratios of BF cirrhosae genome (100, 10, 1, 0.1, 0.01, and 0%, in percentage) were used as the templates. Figure 3C shows that the amplification curve appeared even in detecting mixed templates containing 0.1% of the BF cirrhosae genome. The result was also verified by electrophoresis (Figure 3D), demonstrating that the RALA assay could detect trace amounts of BF cirrhosae in the mixed templates.

### Colorimetric detection of bulbus fritillariae cirrhosae

**FIGURE 4:**
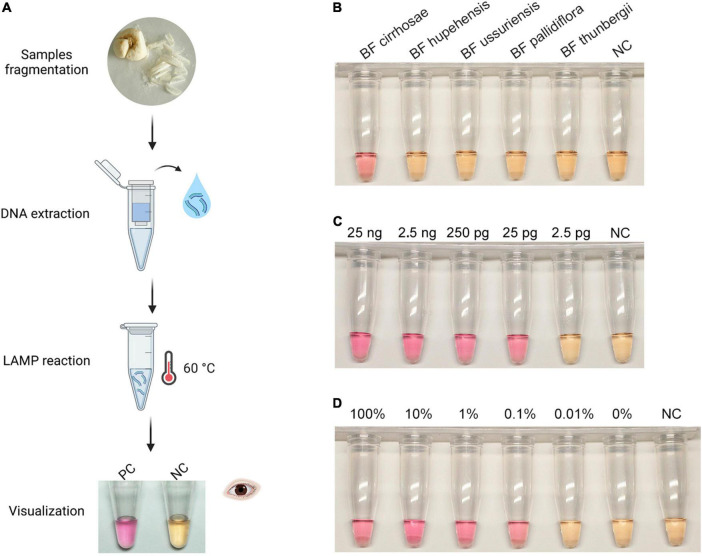
Visualization of the Recombinase Assisted Loop-mediated isothermal DNA Amplification (RALA) amplification. **(A)** Workflow chart: the BF cirrhosae samples were fragmented firstly, and then the genome was extracted by column extraction. The obtained deoxyribonucleic acid samples were then subjected to RALA reaction (including pre-added N-Red) and incubated at 60°C for 80 min to obtain colorimetric detection results. **(B)** Specific colorimetric RALA detection of BF cirrhosae using pre-added N-red. **(C)** Colorimetric analysis of the sensitivity in the range of 25 ng to 2.5 pg. **(D)** Colorimetric detection of BF cirrhosae in mixed templates at varying percentages (100, 10, 1, 0.1, 0.01, 0%).

After the establishment of the real-time RALA assay for BF cirrhosae, we intended to extend its use to a simpler situation of field detection without the need for fluorescence monitoring. Many colorimetric dyes including calcein, phenol red, neutral red (N-red), and so on have been proved to be applicable in realizing on-site colorimetric isothermal detections (Tomita et al., 2008; Tanner et al., 2015). Among these dyes, N-red, with its distinct color changes from yellow to pink when the pH value decreases from 8.0 to 6.4, has been successfully applied in colorimetric LAMP assays (Ding et al., 2019; Wang et al., 2019). Since LAMP and RALA reactions have similar pH changes, we introduced the N-red to the RALA assay to establish colorimetric RALA detection for BF cirrhosae. The colorimetric detection workflow of BF is shown in Figure 4A. Firstly, DNA was extracted from fragmented BF samples by the kit column method, and then the RALA reaction with N-red was carried out. After incubation at 60°C for 80 min, the visual results were obtained. As shown in Figure 4B, the only tube containing the BF cirrhosae templates displayed evident pink color after the incubation. It could be easily discriminated from the yellow color of those tubes containing other non-target samples, suggesting that the N-red could be used in the RALA assay to reflect the amplification results. Then, we investigated the sensitivity of the colorimetric RALA assay. As illustrated in Figure 4C, the color of the tubes containing 25 ng to 2.5 pg of BF cirrhosae genome presented obvious pink, while the color of the rest tubes remained orange-yellow after the incubation, implying that the sensitivity of colorimetric RALA is 200 pg, which was consistent with the real-time assay (Figure 3A). And then we examined the sensitivity of the colorimetric detection method in mixed templates. As shown in Figure 4D, the colorimetric RALA assay could also distinguish as low as 0.1% BF cirrhosae from the mixed samples. It was worth noting that the N-red was preadded to the reaction tube, which could realize one-pot detection and decrease the carry-over contamination. Thus, the colorimetric RALA method could be an appealing choice for field detection of BF cirrhosae.

### Establishment of recombinase assisted loop-mediated isothermal DNA amplification-based identification method for bulbus of *Fritillaria ussuriensis* Maxim., bulbus of *Fritillaria pallidiflora* Schrenk., bulbus of *Fritillaria hupehensis* Hsiao et K. C. Hsia., and bulbus of *Fritillaria thunbergii* Miq.

**FIGURE 5:**
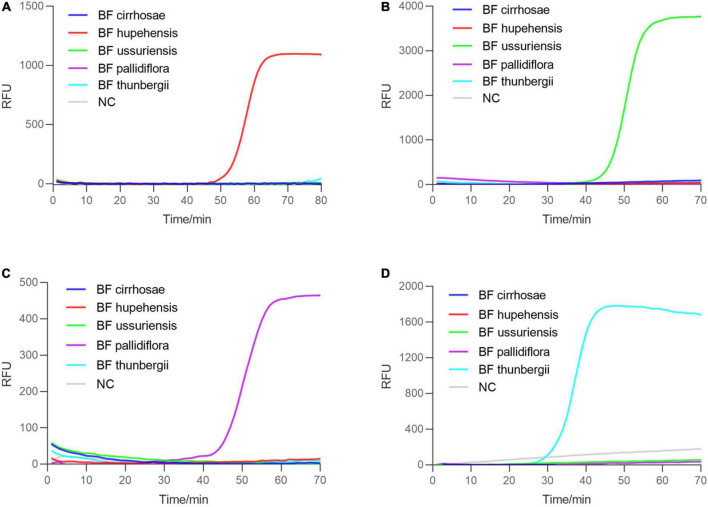
Real-time Recombinase Assisted Loop-mediated isothermal DNA Amplification detection of four BF. Five BF and blank negative control were detected simultaneously with the corresponding specific primers of BF. **(A–D)** The amplification plot using the specific RALA primers for BF hupehensis, BF ussuriensis, BF pallidiflora, and BF thunbergii, respectively.

**FIGURE 6:**
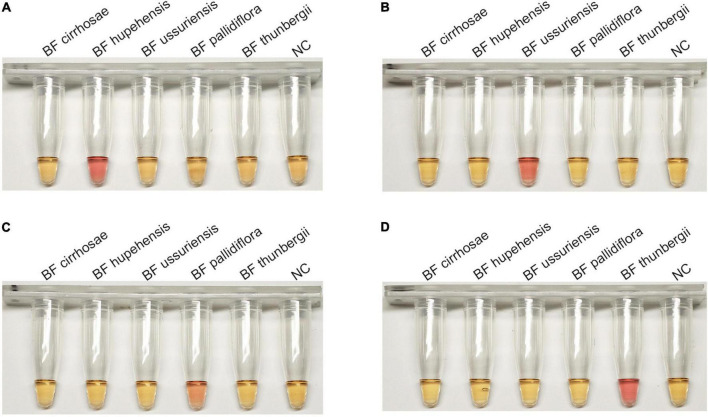
The colorimetric detection of four BF by using N-red reporter in Recombinase Assisted Loop-mediated isothermal DNA Amplification system. **(A)** BF hupehensis. **(B)** BF ussuriensis. **(C)** BF pallidiflora. **(D)** BF thunbergii.

The successful establishment of RALA detection system for BF cirrhosae motivated us to develop the identification method for other common BF including BF ussuriensis (bulbus of *F. ussuriensis* Maxim.), BF pallidiflora (bulbus of *F. pallidiflora* Schrenk), BF hupehensis (bulbus of *F. hupehensis* Hsiao et K. C. Hsia) and BF thunbergii (bulbus of *F. thunbergii* Miq.). The ITS sequences of these four plants were aligned and the specific primer sets for BF ussuriensis and BF pallidiflora were designed (Supplementary Table 2 and Supplementary Figure 1). Due to the high similarity of ITS sequences between BF thunbergii and BF hupehensis, the ITS sequence could not distinguish the two with RALA reaction. Therefore, the RAPD marker p16 genomic sequence (Li et al., 2014; KF934438.1) of BF thunbergii was selected as the candidate target. We designed PCR primers of the P16 gene (Supplementary Table 1) to detect eight BF samples. As shown in Supplementary Figure 4, only BF thunbergii and BF hupehensis were amplified, and their products were sequenced and uploaded to the GenBank (the accession number were ON493814 and ON493815). By comparing the sequencing results, we obtained their specific sites and designed the RALA primers of BF thunbergii and BF hupehensis (Supplementary Figures 5 and 6). The specificity of these primer sets was parallelly determined by detecting all five kinds of BF independently. As shown in Figure 5, each primer set for BF hupehensis, BF ussuriensis, BF pallidiflora, and BF thunbergii showed a positive amplification curve only when amplifying their corresponding templates, indicating the high specificity of these primer sets. Similarly, one-pot colorimetric detection was also evaluated. As shown in Figure 6, the positive amplification tubes, which contained the specific primer set and its corresponding template, presented a color change from yellow to pink, while the amplification tubes containing the unmatched primer-template remained yellow. Therefore, these results demonstrated that the RALA-based identification system is successfully developed to detect all these marketable different BF, providing a universal tool to distinguish different kinds of BF.

## Conclusion

Currently, traditional identified methods based on traits, biological tissue structure and chemical composition are still important means in the identification of crude drug. However, these methods either require expert knowledge about taxonomy or professional personnel and bulky instruments, which are inconvenient to be popularized. Thus, DNA-based molecular detection has been proposed to distinguish herbal medicines from the aspect of genetic, which could be easily applied to discern different herb species, especially the homologous subtypes like BF in this study. At present, the main molecular identification method is PCR. However, PCR reaction relies on the delicate thermal cycling instrument, and the amplification products are often required further analysis by agarose gel electrophoresis to obtain the reports (Qi et al., 2018). Chen et al. compared the detection capabilities of super, general and special DNA barcodes through sequencing analysis, and proved that super barcodes can accurately locate the plant origin of Fritillaria (Chen Q. et al., 2020). Zhu et al. (2021) established a high-throughput rapid detection method for BF cirrhosae and its adulterants by using multiplex ligation-dependent probe amplification and high-resolution melting curve assay. However, these methods need professional operators and precision instruments for detection.

In this study, a RALA based real-time fluorescence identification system is firstly established to identify BF cirrhosae. This system could detect as low as 0.1% of the genuine target in the mixed samples. Moreover, one-pot colorimetric detection could be easily achieved by pre-adding N-red dye. Furthermore, the proposed method was successfully implemented to identify other marketable BF drugs including BF ussuriensis, BF pallidiflora, BF thunbergii, and BF hupehensis. In summary, this RALA-based identification system comprises several advantages: (i) the whole testing can be achieved at a constant temperature, which is less instrument-demanding and cost-efficient; (ii) the high sensitivity enables the method for qualitative and quantitative laboratory testing; (iii) the colorimetric detection could provide a highly useful method in field testing using a simpler heat blocker; (iv) the method could not only distinguish the genuine BF cirrhosae but also determine its possible adulterants, which may complement the isothermal toolbox for BF identification. Therefore, this RALA-based method offers a promising alternative to the molecular identification of BF. As a new isothermal amplification method, RALA combined the merits of recombinase polymerase amplification (RPA) and LAMP. It can realize similar amplification efficiency to LAMP, while requiring less primer sequence. The system of RALA is less complicated than RPA, thus decreasing the cost. Besides, the addition of recombinase could improve the detection specificity of the RALA assay. Moreover, the colorimetric assay developed here could be realized without uncapping operation, which eliminates the false positive caused by carry-over contamination. Despite the advantages above, the method faces some challenges. The first is the primer design of RALA still requires multiple primer sequences, though less complex than LAMP; the second is that RALA carries out under about 60°C, which means a temperature controller is needed, though it is much simpler than PCR-based apparatus. Once overcoming the challenges, we believe that the high practicality of the RALA-based method could be readily extended for the identification of other herbal medicines.

## Data availability statement

The original contributions presented in this study are publicly available. This data can be found here: NCBI, ON493814 and ON493815.

## Author contributions

ZT and RC: conceptualization, supervision, and funding acquisition. YW: methodology, software, validation, investigation, experimental operation, data analysis, drafting, and revision. SD: methodology, experimental guidance, and revision. GC: experimental guidance. JD: investigation. FD: resources and investigation. XH and XC: investigation. All authors contributed to the article and approved the submitted version.

## Abbreviations

BF: Bulbus Fritillariae
BF cirrhosae: Bulbus Fritillariae cirrhosae
RALA: Recombinase Assisted Loop-mediated isothermal DNA Amplification
LAMP: loop-mediated isothermal amplification
ITS: internal transcribed spacer
SGI: SYBR Green I
N-red: Neutral red
BF cirrhosa: bulbus of *Fritillaria cirrhosa* D. Don.
BF unibracteata: bulbus of *Fritilaria unibracteata* Hsiao et K. C. Hsia
BF delavayi: bulbus of *Fritilaria delavayi* Franch.
BF taipaiensis: bulbus of *Fritilaria taipaiensis* P. Y. Li
BF przewalskii: bulbus of *Fritilaria przewalskii* Maxim.
BF wabuensis: bulbus of *Fritilaria unibracteata* Hsiao et K. C. Hsiavar. wabuensis (S. Y. Tanget S. C. Yue) Z. D. Liu, S. Wang et S. C. Chen
BF hupehensis: bulbus of *Fritillaria hupehensis* Hsiao et K. C. Hsia.
BF ussuriensis: bulbus of *Fritillaria ussuriensis* Maxim.
BF pallidiflora: bulbus of *Fritillaria pallidiflora* Schrenk.
BF thunbergia: bulbus of *Fritillaria thunbergii* Miq.
