# Supplementary material for: Real-time fluorescence and colorimetric identification of bulbus fritillariae using recombinase assisted loop-mediated isothermal DNA amplification (RALA)
Source: Front Plant Sci. 2022 Jul 28;13:948879. doi: 10.3389/fpls.2022.948879 (PMC9366889; doi:10.3389/fpls.2022.948879)
Supplement: Supplementary file 1 [file Data_Sheet_1.docx]

**Supplementary information：**

**Real-time Fluorescence and Colorimetric Identification of Bulbus Fritillariae using** **Recombinase Assisted Loop-mediated isothermal DNA Amplification (RALA)**

Yinghua Wei^1,4†^, Sheng Ding^1,4†^, Gangyi Chen^1^, Juan Dong^1^, Feng Du^1^, Xin Huang^1^, Xin Cui^1^, Rong Chen^2,3^* and Zhuo Tang^1^*

^1^ Natural Products Research Center, Chengdu Institute of Biology, Chinese Academy of Sciences, Chengdu 610041, P. R. China;

^2^ School of Ethnic Medicine, Chengdu University of Traditional Chinese Medicine, Chengdu 611137, P. R. China;

^3^ State Key Laboratory of Southwestern Chinese Medicine Resources, The Ministry of Education Key Laboratory of Standardization of Chinese Herbal Medicine, Chengdu University of Traditional Chinese Medicine, Chengdu 611137, China;

^4^ University of Chinese Academy of Sciences, Beijing 100049, P. R. China.

^†^ Yinghua Wei and Sheng Ding contributed equally to this work.

* correspondence:

tangzhuo@cib.ac.cn; chenrong@cdutcm.edu.cn

**Table S1** The PCR primers

| **Primer NO.** | **Name** | **Sequence（5'to3'）** |
| --- | --- | --- |
| BF | F  R | GTAAACGGATGACACCGTGTCG  TTGCCGAGAGTCGTATGGATAGAGA |
| BF thunbergii-P16  (ON493814) | F  R | ACTGGGGGAGAAGTCAAATACTG  GAGGGTATGTGTCGACTACACAG |

**Table S2** Specific RALA primers of BF cirrhosae, BF ussuriensis, BF pallidiflora, BF thunbergii, and BF hupehensis

| **BF Name** | **Accession NO.** | **Primer Name** | **Sequence（5'to3'）** |
| --- | --- | --- | --- |
| BF cirrhosae | MN121633.1 | FIP  BIP  LF | TCCCCCGCAAATCGTGCCC-ACTATGCCCGCCCTGCC GCCGAGGGCACGCCTGC-ACCCGAAGGGTCATTGG  GGAGAGACACGAT |
| BF ussuriensis | KT008197.1 | FIP  BIP  LF  LB | GGCGCTTGGAGGCGATC-GTCGGGCGGACAATTT  GCCTGCGCCAAGGAACA-GTATAGATAGAGAGCGGGCG  GCAGGGCGGCCAAAGA  CCTAAGCGGCGGGGC |
| BF pallidiflora | MN121628.1 | FIP  BIP  LF | CGCAAACCGTGTCCGGAG-CACTG**C**GCTCGCCCT(mismatch T→C）  GGCCTGCGCCAAGGAAC-CGATGGATAGAGATCGAACGT  CGCCCTGAGGCGA |
| BF thunbergii | ON493814 | FIP  BIP  LF | GGTAAGTTCTTGGGGCACATAGTG-GTATGTCTTTCACTGT**CG**TTGG（mismatch A→CG）  TCCTCTAGGTTCCGGATATGATCC-AGAATCTTTCAAGGACTGCTAG  TCCTGCACAGACA |
| BF hupehensis | ON493815 | FIP  BIP  LF | CGCAGATCCAGCGCAAAC-TTCTTCCACTCAGTAC  TCAAGGTTCTGGATATGATCCTCC-GATCTTTCAAGGGCTG  GTCCC**G**GAACAGT（mismatch A→G） |

Note: Red base is the artificial mutation or insertion base.

**Table S3** Candidate specific RALA primers for BF cirrhosae

| **Primer NO.** | **Name** | **Sequence（5'to3'）** |
| --- | --- | --- |
| BF cirrhosae-1 | FIP  BIP  LF  LB | TCCCCCGCAAATCGTGCCC-ACTATGCCCGCCCTGCCCG  TGCCTGGGCGTCACGCCTTG-GCATCCGTGACCGCACCC  AGAGACACGATGCGAGGT  CTCCGTGCCCAATGACCCT |
| BF cirrhosae-2 | FIP  BIP  LF | TCCCCCGCAAATCGTGCCC-ACTATGCCCGCCCTGCCCG  TGCCTGGGCGTCACGCCTTG-GCATCCGTGACCGCACCC  AGAGAGCACGATG |
| BF cirrhosae-3 | FIP  BIP  LF  LB | CCCCGCAAATCGTGCCC-TATGCCCGCCCTGCC  CCTGGGCGTCACGCCTT-ATCCGTGACCGCACCC  AGAGACACGATGCGAGGT  CTCCGTGCCCAATGACCCT |
| BF cirrhosae-4 | FIP  BIP  LF  LB | CCCCGCAAATCGTGCCG-CCCGCCCTGCCC  CTGGGCGTCACGCCTTG-ATCCGTGACCGCACC  AGAGACACGATGCGAGGT  CCGTGCCCAATGACCCT |
| BF cirrhosae-5 | FIP  BIP  LF  LB | GCAAATCGTGCCCGGAGA-CCCGCCCTGCCC  GCCTGCCTGGGCGT-CGCACCCGAAGGGTC  GACACGATGCGAGGTCC  TTCGCTCCGTGCCCAAT |
| BF cirrhosae-6 | FIP  BIP  LF | TCCCCCGCAAATCGTGCCC-ACTATGCCCGCCCTGCC GCCGAGGGCACGCCTGC-ACCCGAAGGGTCATTGG  GGAGAGACACGAT |


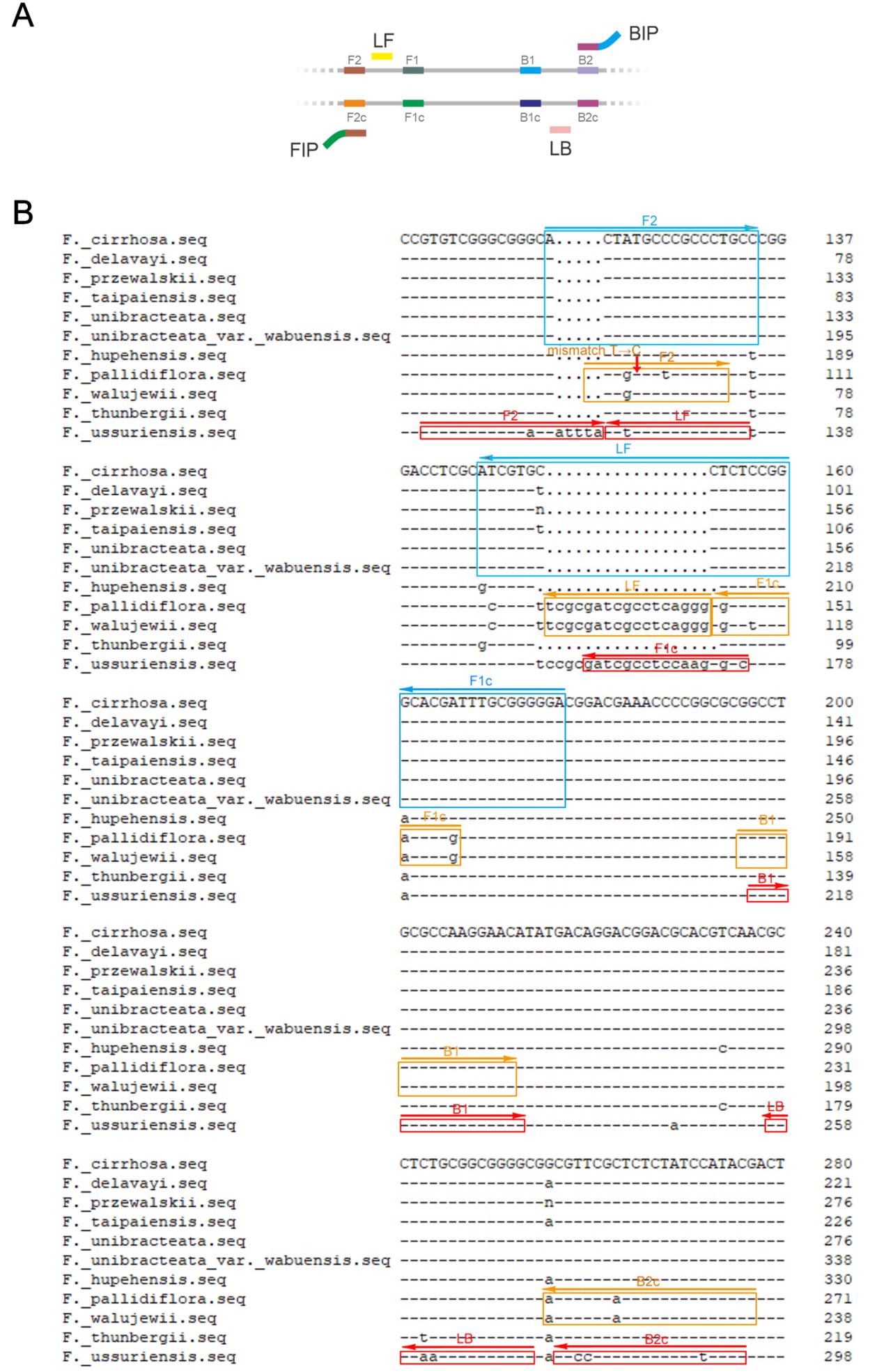


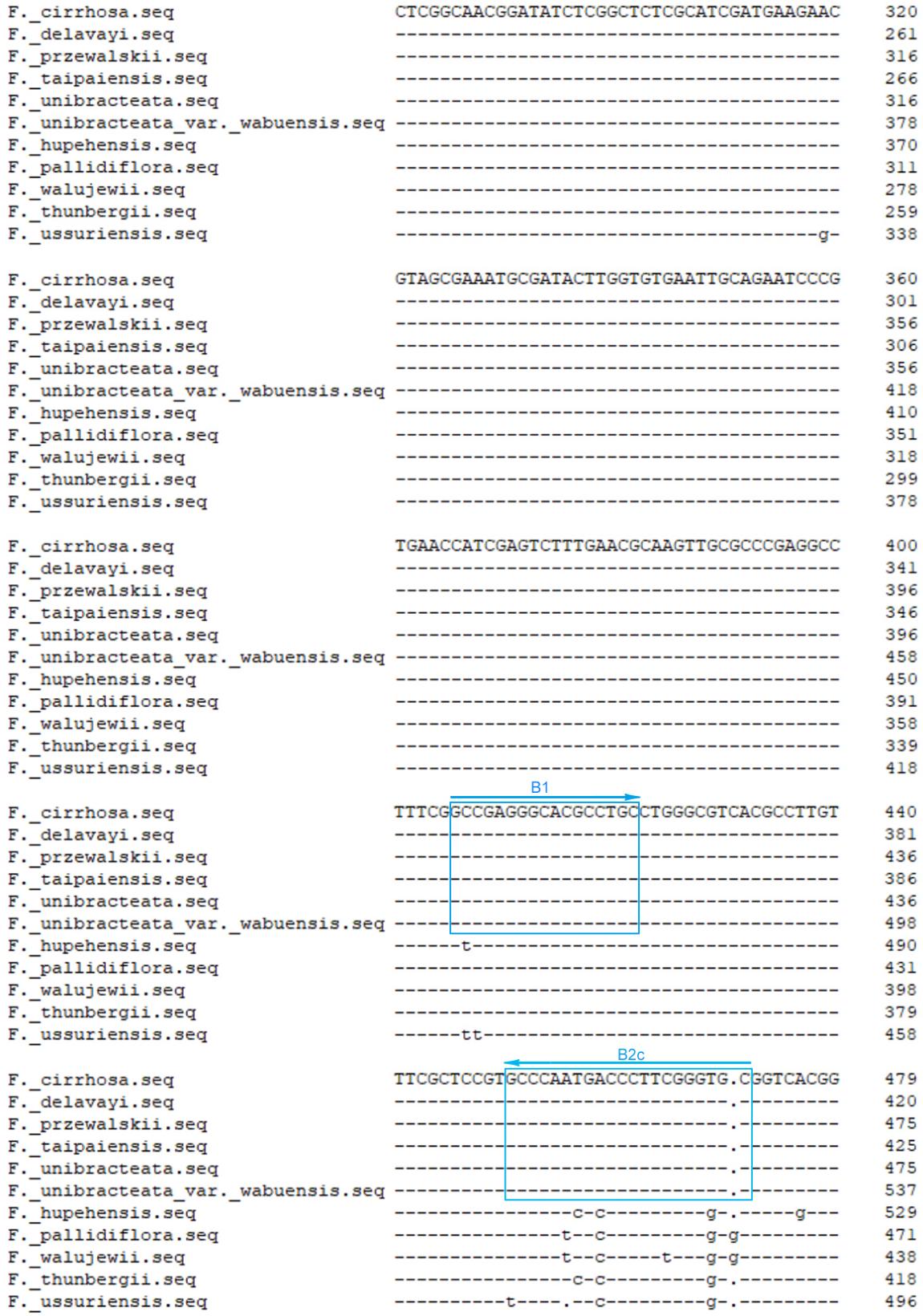


**Figure S1** (A) Schematic diagram of RALA primer design; (B) By comparing ITS sequences of BF cirrhosae (including *F. cirrhosa* (MN121633.1), *F. delavayi* (MN184748.1), *F. przewalskii* (MF083552.1), *F. taipaiensis* (MH711398.1), *F. unibracteata* (MG525330.1), *F. unibracteata var. wabuensis* (KX669649.1)), BF pallidiflora (including F. *pallidiflora* (HQ010405.1) and *F. walujewii* (KP712008.1)), BF ussuriensis (DQ191622.1), BF thunbergii (HQ448863.1), and BF hupehensis (KF906203.1), specific sites of each BF drugs in ITS sequence were obtained, and RALA primers of BF cirrhosae, BF pallidiflora and BF ussuriensis were designed at these sites.


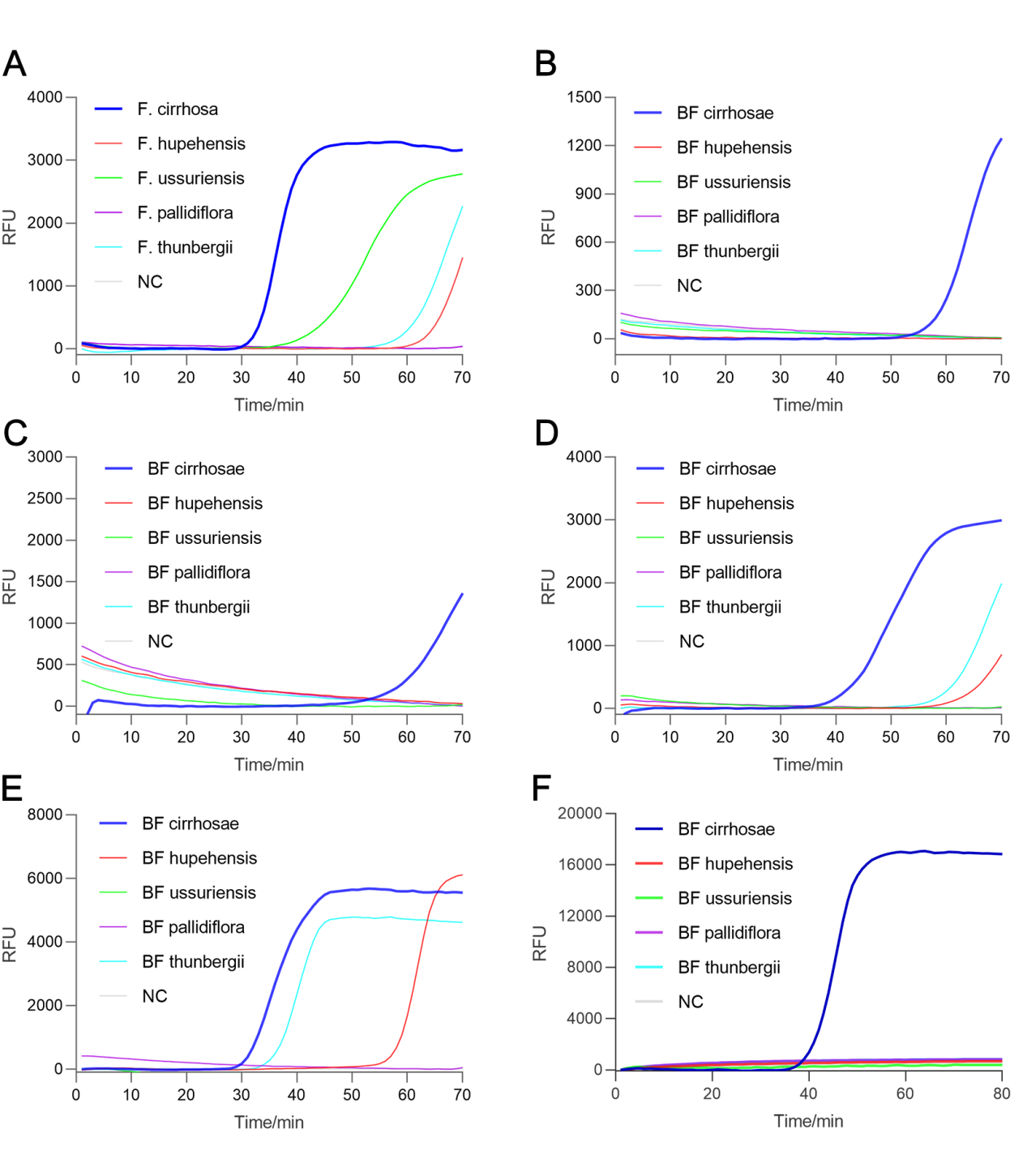


**Figure S2** Screening of RALA specific primers for BF cirrhosae. (A-F) The amplification plot using the RALA primers of BF cirrhosae-1, BF cirrhosae-2, BF cirrhosae-3, BF cirrhosae-4, BF cirrhosae-5, and BF cirrhosae-6 to amplify the genomic DNA of various BF drugs.


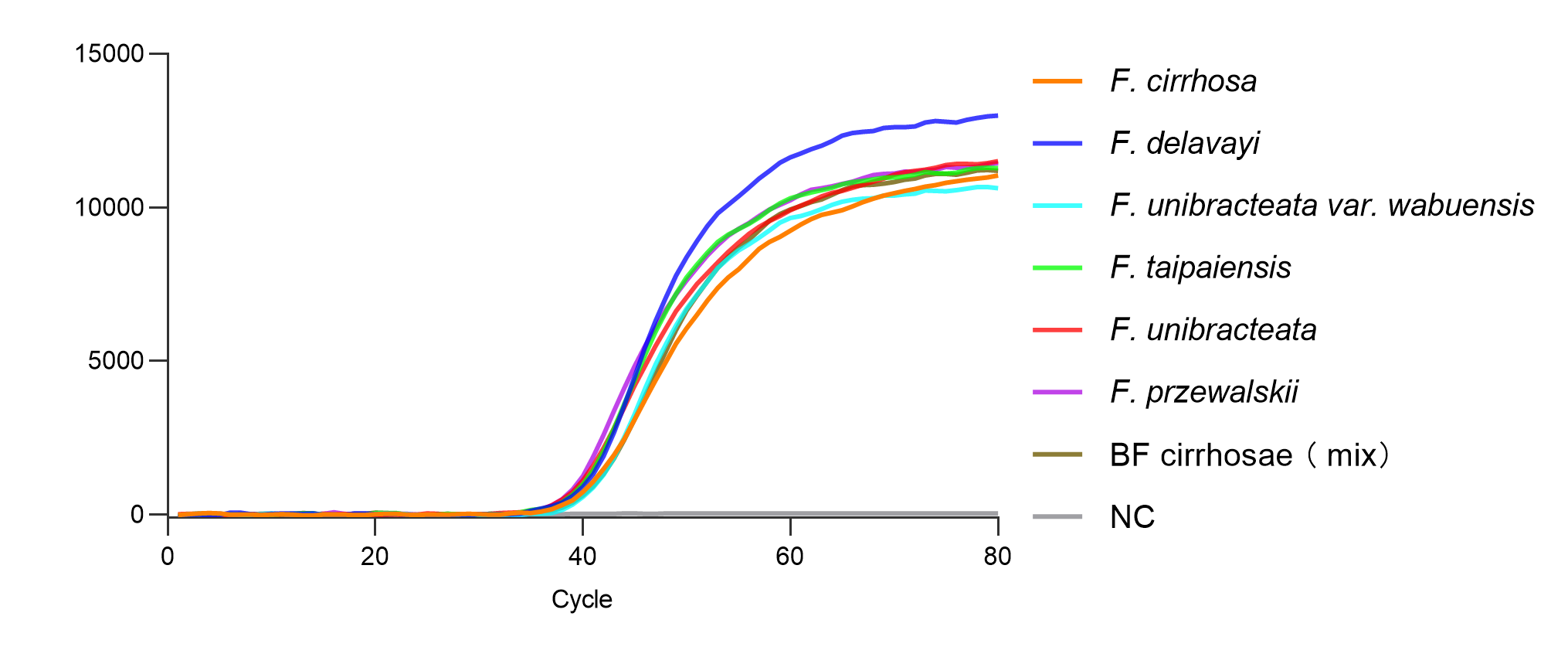


**Figure S****3** The RALA amplification curves results of the genomic DNA of six kinds of BF cirrhosae and the equal proportion mixed samples of the six kinds of BF cirrhosae.


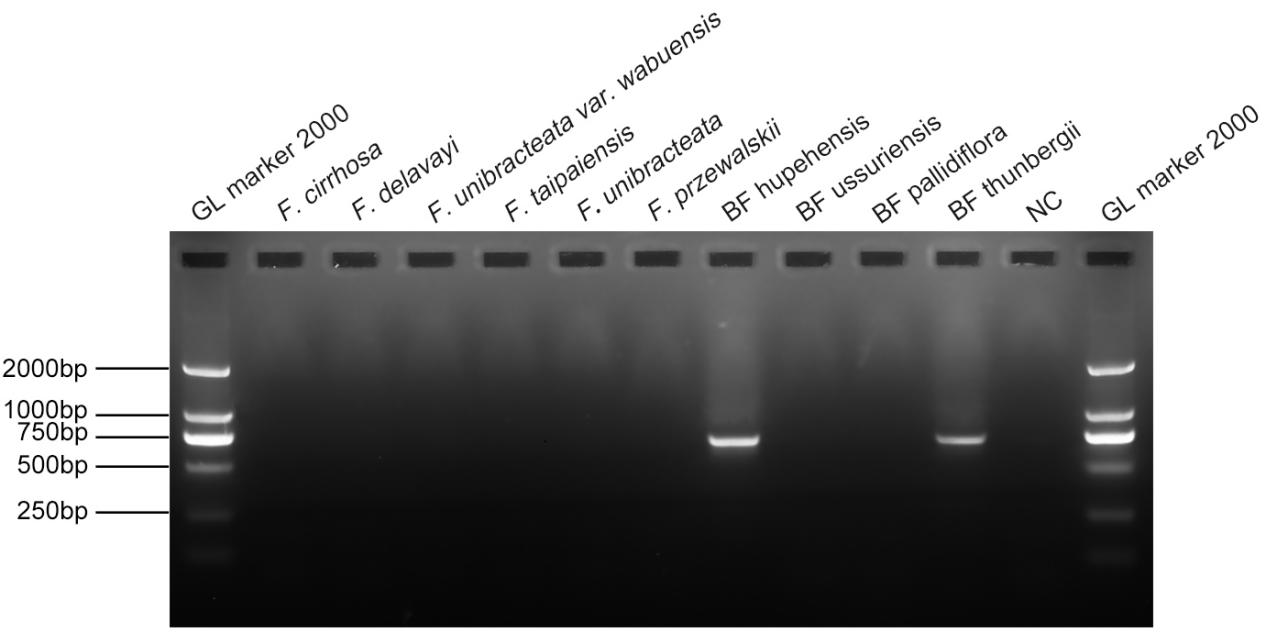


**Figure S4** Electrophoresis analysis of PCR products amplified by BF thunbergii-P16 primer set.


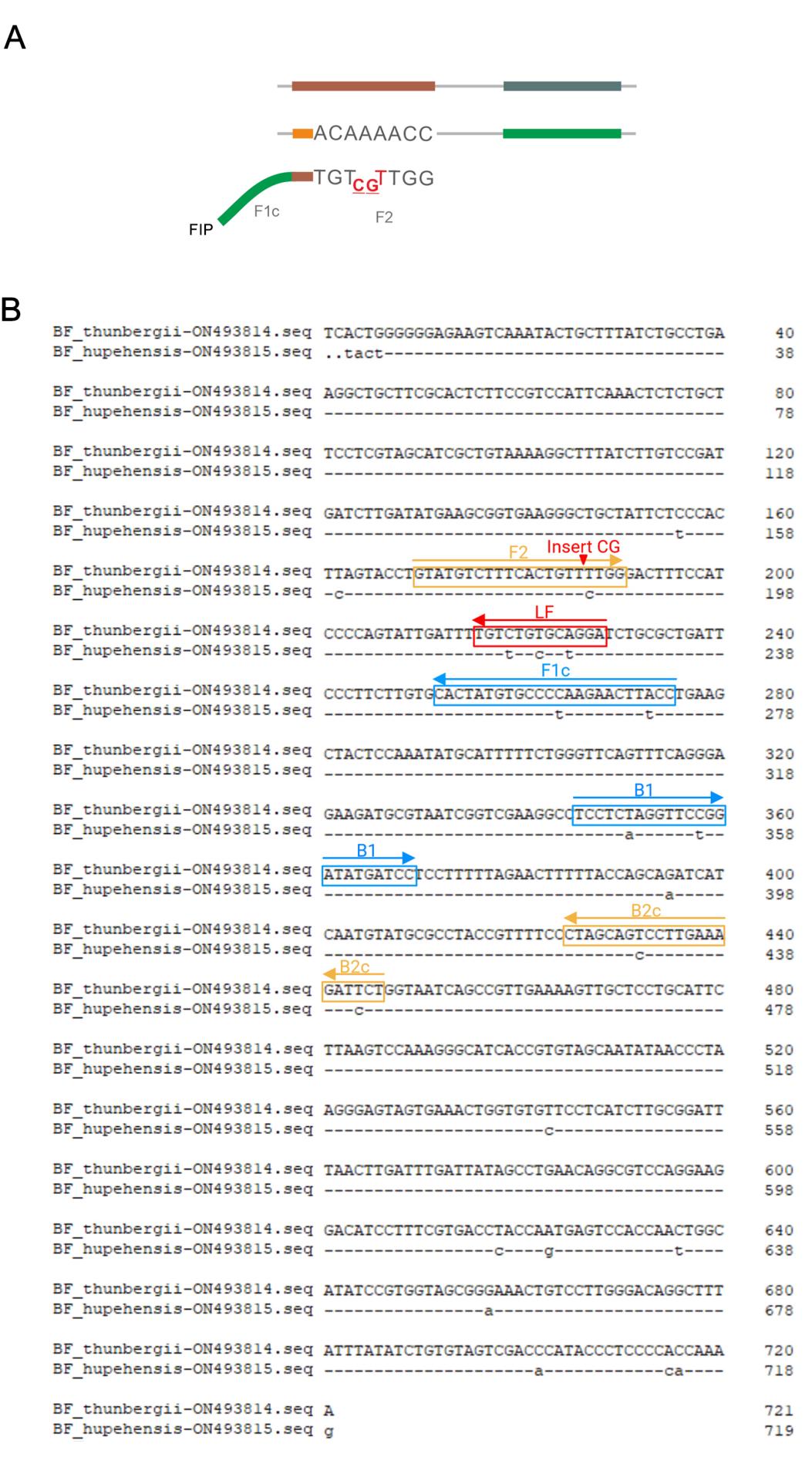


**Figure S5** Primer design and sequence alignment of the P16 gene of BF thunbeigii (GenBank accession number ON493814) and BF hupehensis (GenBank accession number ON493815). (A) The FIP primer of BF thunbeigii containing mismatched bases which were designed to improve the specificity; (B) The specific RALA primer site of BF thunbeigii.

**
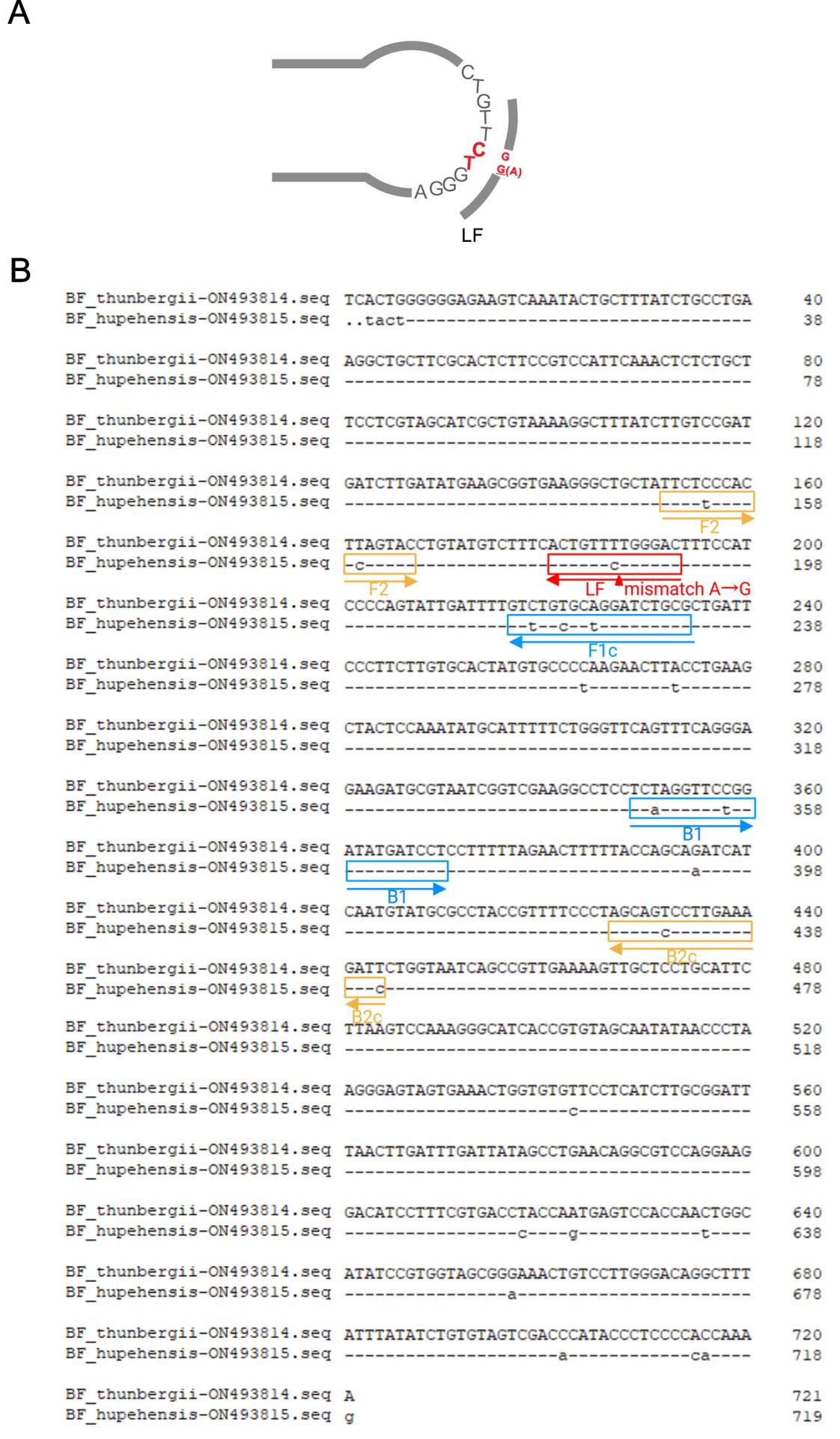
**

**Figure S6** (A) The mismatch designed in loop primer of BF hupehensis; (B) The specific RALA primers for BF hupehensis.
